# Electrically evoked compound action potentials in cochlear implant users with preoperative residual hearing

**DOI:** 10.3389/fnhum.2023.1125747

**Published:** 2023-10-02

**Authors:** Tim Liebscher, Joachim Hornung, Ulrich Hoppe

**Affiliations:** ENT-Clinic, Department of Audiology, Friedrich-Alexander-Universität Erlangen-Nürnberg (FAU), Erlangen, Germany

**Keywords:** cochlear implant, residual hearing, electrically evoked compound action potential, pure-tone audiometry, peripheral neural survival, nerve-electrode interface, outcome prediction

## Abstract

**Introduction:**

Residual hearing in cochlear implant (CI) candidates requires the functional integrity of the nerve in particular regions of the cochlea. Nerve activity can be elicited as electrically evoked compound action potentials (ECAP) after cochlear implantation. We hypothesize that ECAP thresholds depend on preoperative residual hearing ability.

**Materials and methods:**

In a retrospective study, we analyzed 84 adult cochlear implant users who had received a Nucleus^®^ CI632 Slim Modiolar Electrode and who preoperatively had had residual hearing. Inclusion criteria were severe to profound hearing loss with preoperative measurable hearing in the ear to receive the implant, postlingual hearing loss, German as native language and correct placement of the electrode, inserted completely into the scala tympani. Electrically evoked compound action potential (ECAP) was recorded intraoperatively. The angular insertion was measured for each electrode contact from postoperative computed tomography to estimate the corresponding spiral ganglion frequency. Pure-tone audiometry and allocated ECAP thresholds were tested to investigate possible correlation.

**Results:**

The average of hearing thresholds, tested at 0.5, 1, 2, and 4 kHz (4FPTA) was 82 ± 18 (range 47–129) dB HL. The success rate for recording ECAP thresholds was 96.9%. For all comparable pure-tone frequencies (1, 2, 4, and 8 kHz), there was significant correlation between preoperative hearing levels and intraoperative ECAP thresholds (*p* < 0.001). Higher hearing thresholds are associated with increased ECAP thresholds.

**Conclusion:**

In CI candidates with adequate residual hearing, intraoperative electrophysiological measurement records lower thresholds. This outcome may be explained by the neural survival density of the peripheral system, with less neural degeneration.

## 1. Introduction

A cochlear implant (CI) can restore the ability to hear sounds and to recognize speech in patients with severe-to-profound hearing loss and even in cases with complete deafness. The CI consists of an electrode array that is positioned directly in the cochlear and can electrically stimulate the auditory nerve. After CI surgery and initial activation, with increasing CI experience the auditory performance with CI generally improves (Krueger et al., 2008). However, the outcome is influenced by various different factors, e.g., etiology, duration of auditory deprivation, grade of hearing loss, residual speech recognition and patient’s age at implantation (Friedland et al., 2003; Blamey et al., 2012; Lazard et al., 2012). There is still a large variability in CI outcome.

In a recent study, Rieck et al. (2023) demonstrated in a large CI cohort of 538 adult ears a median postoperative monosyllabic word recognition score of 75% in quiet at 70 dB SPL. While half of all ears achieved a recognition score between 55 and 85%, one quarter defined as ‘good performers’ scored between 85 and 100% and the remaining quarter of ‘poor performers’ scored only between 10 and 55%.

Besides the patients’ etiology, the final positioning of the CI electrode array can also influence the postoperative hearing outcome. The excitation of a population of auditory nerve fibers by the stimulation of a single electrode is defined as “electrode–neuron interface” (Bierer, 2010; He et al., 2017). E.g., a larger distance from the source electrode to the targeted neurons will eventually need higher stimulation to evoke a hearing perception. Higher stimulation levels will widen the electrical field, which can lead to a broader spread of excitation and also a greater degree of channel interaction (Bierer, 2010). Hence, the positioning of the CI electrode with regard to insertion depth (completely vs. incompletely inserted), scala vestibuli (SV) placement or malpositioning of the electrode array (tip foldover, kinked electrode) can alter cochlear coverage and the distance between electrode contacts and targeted neurons, and extend place-pitch changes (Aschendorff et al., 2017).

In order to estimate the CI outcome during CI diagnosis, preoperatively measured speech recognition scores under aided and unaided conditions can be used. Earlier studies have demonstrated that the preoperative maximum word recognition score (WRS_max_) in subjects with unaided speech recognition correlates positively with postoperative hearing performance with a CI (McRackan et al., 2018; Hoppe et al., 2019). Hoppe et al. (2019) explain this by reference to the fact that the WRS_max_ shows the effects of “reduced temporal and spectral resolution in the entire auditory system” and also reflects the individual’s neuronal processing capacity. Furthermore, they showed that the WRS_max_ can be used as a minimum estimator for postoperative speech recognition with the CI system.

In addition to speech audiometry, hearing levels are objectified through pure-tone audiometry (PTA), which reflects neural survival rate of the peripheral system. Subjects with substantial low-frequency residual hearing can even use electric acoustic stimulation (EAS), which may increase hearing performance with a CI later on (Incerti et al., 2013). Typically, the PTA in CI candidates is used for diagnosis and determination of indication; later in CI users it is employed to determine the residual hearing to adjust acoustic component in EAS, if applicable. In comparison with the pre- and post-CI status, PTA is mostly used in patients who use electric acoustic stimulation (EAS) as a measure of hearing preservation that describes the amount of additional sensorineural hearing loss due to the trauma induced during CI surgery.

In recent years electrocochleography (ECoG) has gained increasing importance as a measuring tool in CIs. ECoG records the electrical potentials which are generated in the inner ear and auditory nerve after an acoustic stimulus is presented. This allows to objectively characterize the function of the peripheral auditory system and to acoustic hearing. ECoG measures can be recorded extracochlear using a recording electrode placed at the promontory, the stapes, or the tympanic membrane. Modern CI systems can also use the intracochlear electrodes as recording electrode (Haumann et al., 2019; Kim, 2020). Intraoperatively, ECoG can be used to monitor the status of acoustic hearing during CI surgery. This real-time information during CI insertion can help the surgeon to reduce insertion trauma and thereby preserve residual hearing. In CI users with postoperative residual acoustic hearing, ECoG has also been used to monitor the status of acoustic hearing preservation. Studies have shown that intraoperative ECoG can be recorded in most CI implanted subjects (Choudhury et al., 2012; Fitzpatrick et al., 2014) and some study groups present moderate correlation of intraoperative ECoG responses with the postoperative CI outcome (Gifford et al., 2013; Fitzpatrick et al., 2014). Their prognostic value with regard to the hearing preservation is still inconsistent (Kim, 2020).

Even though ECoG measures can provide important information of the auditory periphery, in most CI patients responses can only be recorded reliably until 1,000 Hz (Choudhury et al., 2012; Fitzpatrick et al., 2014), since the hair cell loss in the basal areas is more severe. Also, the measurement setup and analysis to extract relevant ECoG variables is quite complex (Fontenot et al., 2019). Therefore, this technology is not yet part of the daily routine in most CI clinics.

The majority of today’s CI candidates – even if not regarded as EAS candidates – have preoperative hearing thresholds (Hoppe et al., 2015; Holder et al., 2018; Hey et al., 2020) within the limits of standard clinical audiometers. Depending on the audiometer and headphone output levels, hearing levels up to 130 dB HL can be confirmed. Typically, in hearing-impaired subjects, hearing levels at higher frequencies are poorer than at low frequencies, which approach or go beyond the audiometers’ output levels (Hoppe et al., 2015; Wu et al., 2021). According to the tonotopic organization of the cochlea, these hearing levels depend on survival rate of hair cells and neural structures at certain regions on the basilar membrane. Hence, electrophysiological responses from particular intracochlear positions should correlate with hearing thresholds.

**Figure 1:**
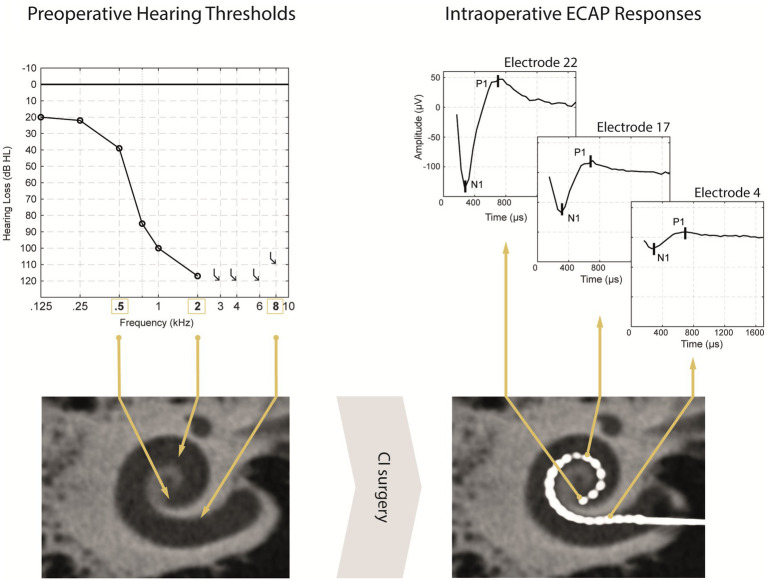
Simplified illustration of a participants pre- and postoperative diagnostic data. Preoperative unaided pure-tone audiometric thresholds with the estimated physiological place-frequency (exemplary for PTA frequencies: 0.5, 2, and 8 kHz) displayed in the preoperative DVT scan (left panel). After CI surgery (right panel), the postoperative DVT scan shows a regularly placed CI electrode array with the corresponding electrode contacts EL22, E17 and E4 positioned at the physiological place-frequency of 0.5, 2, and 8 kHz, respectively. ECAP responses for the same stimulation level differ in magnitude depending of stimulation site from apical to basal electrodes.

After the implantation of a CI, responses of the auditory nerve can be recorded intracochlearly by using electrically evoked compound action potentials (ECAPs). In contrast to the acoustically evoked ECoG measures, ECAPs are evoked by an electrical stimulus. The ECAP response typically shows a biphasic morphology with one negative peak N_1_ between 0.2 and 0.4 ms and one positive peak P_1_ between 0.6 and 0.8 ms after the stimulus onset (Brown et al., 1990; He et al., 2017). The ECAP amplitude is determined by the difference in voltage between the N_1_ and P_1_ (Figure 1). Typically, the amplitude increases as the stimulation intensity is increased. This correlation is shown by the amplitude growth function. ECAP measures can also reveal the ECAP threshold (T-ECAP), which is roughly defined as the minimum of electrical charge that produces an ECAP response.

ECAP responses have already been investigated in various studies in animal models and human CI users. Animal studies have shown a relationship between ECAP responses and the number of surviving spiral ganglion cells (SGCs; Prado-Guitierrez et al., 2006; Ramekers et al., 2014). The slope of the ECAP amplitude growth function in normal-hearing compared with deafened guinea pigs was steeper and the overall responses were increased, and this accorded with the results of histological analysis. The deafened group had a reduced packing density of SGCs, showing an overall SGC degeneration and reduced neural survival of the peripheral auditory system (Ramekers et al., 2014).

In human CI recipients ECAPs are an established tool intraoperatively to monitor and verify the electrode-nerve interface and postoperatively to assist the CI fitting process. In individual cases, ECAP thresholds might be used in clinical programming to set behavioral stimulation levels; but so far studies reported only mixed results with regard to correlation of both parameters (He et al., 2017; de Vos et al., 2018). In addition, ECAPs can detect malpositionings such as tip foldover (Grolman et al., 2009; Müller et al., 2021) or electrode translocations (Mittmann et al., 2015; Liebscher et al., 2021).

Since ECAP thresholds can be recorded in almost all CI users (about 95% of the electrodes; Van Dijk et al., 2007; Müller et al., 2015; Hey et al., 2020; Liebscher et al., 2021), their absence can indicate pathological cases (Müller et al., 2015). Furthermore, other sophisticated ECAP paradigms as spread of excitation measurements (Cohen et al., 2003) were used to record electrode interaction. In addition, it was shown that variation of the inter-phase gap correlates with psycho-electric parameters (Schvartz-Leyzac et al., 2020; Brochier et al., 2021). It can be assumed that these particular measures are more suitable for specific tasks. Unfortunately, no data for large number of subjects exist. In most clinical standard routines only ECAP thresholds are performed. The easy-to-use automated T-ECAP algorithms are an established and transparent measuring tool also used in CI research tasks.

The development of clinical tools to better estimate speech perception outcomes prior to cochlear implantation could provide benefit to CI users. So far, only an approximate prediction can be made in certain patient groups with substantial preoperative speech recognition scores (Hoppe et al., 2019, 2021). In subjects with insufficient preoperative speech comprehension, adequate information about the intracochlear neuronal health status is not available. However, we may hypothesize that in subjects with residual hearing the PTA can be used to reflect the health status of auditory nerve fibers (Figure 1).

The study of Nassiri et al. (2019) showed significant differences in ECAP measures in a large number of CI recipients with and without residual hearing. Residual hearing was defined as unaided air conduction threshold ≤90 dB HL at 250 Hz at CI activation. Their data shows throughout apical and medial electrodes significant lager ECAP amplitudes in patients who had preserved low-frequency acoustic hearing. Additionally, T-ECAPs at apical electrodes were significantly higher in subjects without residual hearing. This study demonstrates that there is an association between ECAP measurements and low-residual hearing at 250 Hz.

However, this study included various CI manufacturers with different electrode types; along with straight lateral wall electrode arrays and pre-curved modiolar hugging electrode arrays. They did not report data with regard to the electrode’s type, length and insertion depth or scalar positioning. Therefore, it is not clear where each electrode contact is actually placed in the cochlea and which cochlear place pitch is stimulated individually. E.g., “apical” electrodes might correspond to very different intracochlear regions in-between patients. Unfortunately, residual hearing at 250 Hz was only used for separating both patient groups with regard to residual hearing; no other frequencies greater than 250 Hz were investigated. Since most electrode arrays do not cover this most apical cochlear region (Landsberger et al., 2015), PTA data from higher frequencies are of interest.

The aim of this study was to determine whether there is a correlation between preoperative PTA data and intraoperative ECAP threshold measurements. Therefore, we analyzed preoperative hearing levels at various frequencies (spaced in octaves) and investigated their correlation with the corresponding individual, anatomical SG frequency maps. On the basis of our clinical experience, we hypothesize that subjects with low PTAs will achieve lower T-ECAPs, since there are more healthy auditory nerve fibers to respond to and the neural degeneration is less severe.

## 2. Materials and methods

### 2.1. Study design

This was a retrospective study which included adult subjects with valid pure-tone thresholds before CI implantation. Preoperative audiometric and speech measurements were acquired within the CI candidacy screening procedure. Objective T-ECAP measurements were conducted intraoperatively during CI implantation.

All subjects agreed to the use of their data in this study as part of a general declaration of consent to clinical research (Ethics Committee approval no. 162_17 Bc Erlangen).

### 2.2. Subjects

Participants were native German speakers and at least 18 years of age at the time of implantation. Onset of severe to profound hearing loss had to be postlingual. All subjects received a Slim Modiolar electrode CI632 (Cochlear Ltd., Sydney, NSW, Australia). Additional inclusion criteria were: fully inserted electrodes with complete scala tympani (ST) positioning. Exclusion criteria were: tip foldover of the electrode; reimplantation or reinsertion of the electrode; abnormalities of the cochlea or of the auditory nerve; cognitive disorders which prevent undergoing audiometric assessment.

The subject group consisted of 42 females and 42 males; their age at time of implantation ranged from 25 to 85 years, with a mean (±standard deviation, SD) of 65 ± 13 years. Of the 84 subjects, 38 received their CI on the left and 46 on the right side. Mean ± SD duration of deafness was 12 ± 13 (range: 1–59) years. Demographic data are shown in Table 1.

**Table 1:** Demographic and CI-related characteristics of the subjects including etiologies, preoperative audiometry, cochlear parameters, surgical approach, and insertion depth angles.

Subjects	
Number	*N* = 84
Gender (female/male)	42/42
Side (left/right)	38/46
Duration of deafness (years)	Mean ± SD: 12 ± 13 (range: 1–59)
Age at implantation (years)	Mean: 65 ± 13 (range: 25–85)
Etiologies	
Cholesteatoma	*n* = 0 (0%)
Chronic otitis media	*n* = 6 (7.1%)
Familial	*n* = 4 (4.8%)
Infection	*n* = 3 (3.6%)
Medicinal	*n* = 2 (2.4%)
Menière’s disease	*n* = 8 (9.5%)
Meningitis	*n* = 2 (2.4%)
Noise	*n* = 1 (1.2%)
Otosclerosis	*n* = 0 (0%)
Sudden hearing loss	*n* = 25 (29.8%)
Syndromal	*n* = 3 (3.6%)
Trauma	*n* = 3 (3.6%)
Tumor	*n* = 0 (0%)
Unknown	*n* = 27 (32.1%)
Preoperative audiometry	Median ± SD (range)
4FPTA (dB HL)	82 ± 18 (47–129)
PT_.125kHz_ (dB HL)	58 ± 21 (8–85)
PT_.25kHz_ (dB HL)	63 ± 22 (10–105)
PT_.5kHz_ (dB HL)	71 ± 18 (10–117)
PT_1kHz_ (dB HL)	80 ± 19 (40–140)
PT_2kHz_ (dB HL)	87 ± 25 (55–140)
PT_4kHz_ (dB HL)	94 ± 28 (45–140)
PT_8kHz_ (dB HL)	110 ± 16 (28–110)
Monosyllabic score @ 65 dB SPL with HA (%)	5 ± 18 (0–99)
Maximum monosyllabic score WRS_max_ (%)	35 ± 26 (0–100)
Anatomical data and electrode positioning	
Cochlear diameter A (mm)	Mean: 9.0 ± 0.5 (range: 8.0–10.0)
Insertion angle most apical electrode (°)	Mean: 380 ± 24.2 (range: 315–425)
Insertion angle most basal electrode (°)	Mean: 9.4 ± 5.2 (range: 1–25)
Medial-lateral position MP	Mean: 0.62 ± 0.03 (range: 0.53–0.70)
Surgical approach	
Round window	*n* = 77
Round window with cochleostomy	*n* = 7

### 2.3. Tone and speech audiometry

Subjects underwent an audiological test battery during the CI assessment. The unaided pure-tone audiometric threshold for octave-spaced frequencies from 125 Hz to 8 kHz were obtained by using air-conduction headphones (DT48; beyerdynamic, Heilbronn, Germany) and the AT 1000 audiometer (Auritec Medizindiagnostische Systeme GmbH, Hamburg, Germany) which was calibrated according to the DIN EN 60318 standard. The contralateral ear was masked appropriately. The pure-tone average threshold (4FPTA) was calculated from frequency thresholds: 0.5, 1, 2, and 4 kHz. If a pure tone was “not heard” at the maximum output level of the audiometer, the subject’s hearing loss was estimated as the maximum output level plus 10 dB.

Word recognition score (WRS) for phonemically balanced monosyllabic words (DIN 45621-1:1995-08, 1995) under unaided conditions was recorded. The headphone presentation level was increased until the maximum score of 100% was reached. Otherwise the maximum score achievable (WRS_max_) that was still below the patient’s loudness level of discomfort was noted (Hoppe et al., 2019).

### 2.4. CI surgery

All CI surgical procedures were performed by the same experienced surgeon according to the manufacturer’s guidelines (Cochlear Ltd, 2020) by the soft-surgery technique. The implantation was carried out by round-window insertion (*N* = 77) or round-window enlargement (*N* = 7). ECAP data were recorded intraoperatively after insertion and closure of the round window opening.

Plain X-ray imaging was used intraoperatively to ensure that no electrodes were inserted incorrectly (e.g., with tip foldover), and digital volume tomography (DVT) was performed within the 2 days after surgery in order to verify in detail that the intracochlear electrode positioning was correct.

### 2.5. Imaging analysis

**Figure 2:**
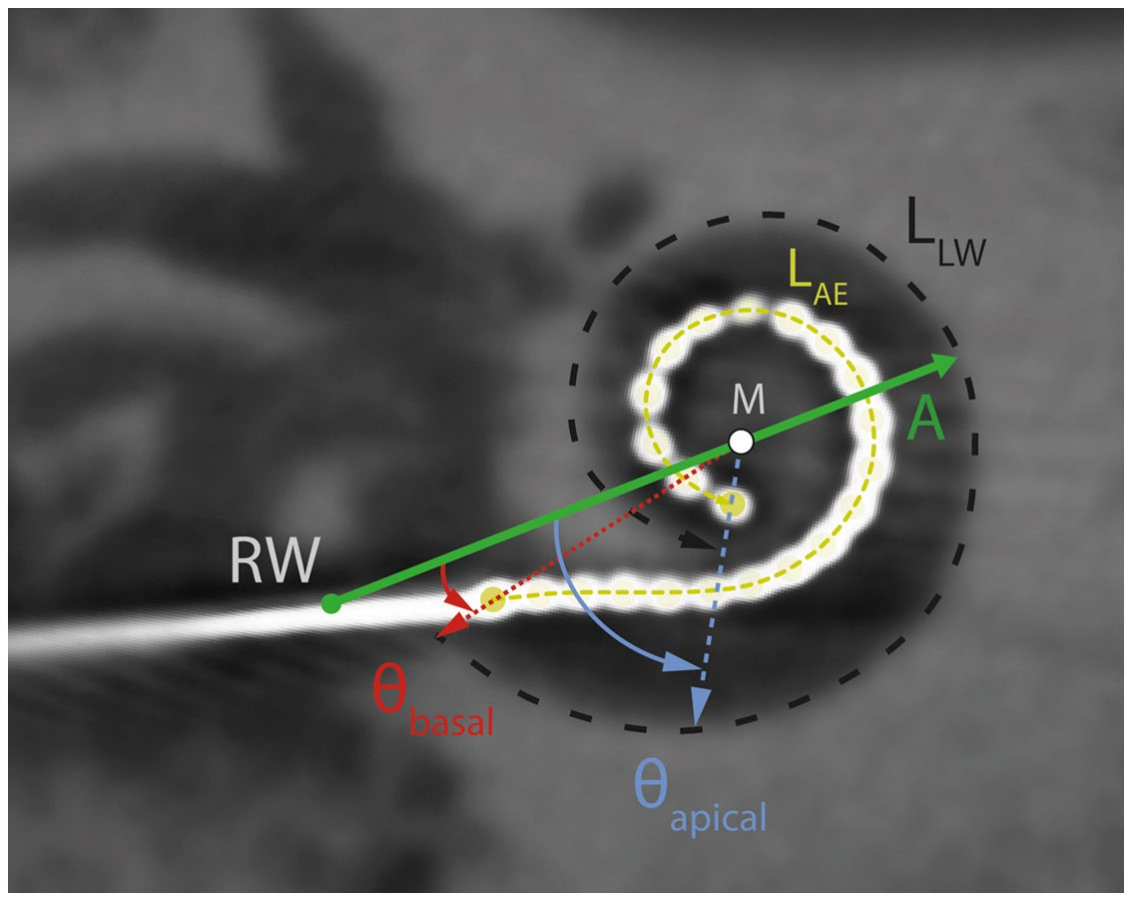
DVT scan in “cochlear view.” Schematic illustration of the cochlear diameter (A; green horizontal line), lateral wall length (L_LW_, black dashed line), active electrode length (L_AE_, yellow dashed line), insertion depth angles at electrode contacts E22 (θ_apical_) and EL1 (θ_basal_), modiolus (M) and round window (RW).

Postoperative DVT scans were analyzed in “cochlear view” (Cohen et al., 1996; Xu et al., 2000) with regard to scalar positioning of electrodes, cochlear size (diameter A), insertion depth angle for the most apical electrode E22 (θ_apical_) and most basal electrode E1 (θ_basal_). Diameter A was measured as the distance between the round window and the outer wall passing through the modiolus, while the insertion depth angles were calculated relative to the reference line drawn between the round window and the modiolus (Figure 2). As described by Aschendorff et al. (2017) the medial-lateral position (MP) can be calculated as the ratio of the active electrode length (L_AE_) according to the manufacturer’s specifications (CI632: 13.4 mm, Cochlear Ltd., Macquarie University, Australia) and the length of the outer wall (L_LW_) for the matching angular insertion depth according to Escudé et al. (2006).

-
$$L_{LW}=2.62A\times \log_{e}\left(1.0+\frac{\theta_{\mathrm{apical}}}{235}\right)-2.62A\times \log_{e}\left(1.0+\frac{\theta_{\mathrm{basal}}}{235}\right)$$
-
$$MP=\frac{L_{AE}}{L_{LW}}$$

The MP describes the average proximity of the electrode contacts to the modiolus. A large MP (up to 1) corresponds to an electrode array that is placed laterally (comparable to straight electrodes), while a small MP describes an electrode array that is very close to the modiolus.

### 2.6. Anatomical frequency allocation

In order to analyze the ECAP responses in relation to the actual cochlear place pitch, the frequency allocation for each electrode’s insertion angle was calculated.

The remaining 20 insertion depth angles between θ_apical_ and θ_basal_ were estimated by linear interpolation for each subject. For each electrode contact, the spiral ganglion frequency was determined according to the findings of Stakhovskaya et al. (2007). Thus, the angular location of the electrode contact corresponds to the tonotopic map of the spiral ganglion cells and to the tonotopic coding of the cochlea. The spiral ganglion map was used, because we investigated only perimodiolar electrode arrays, i.e., those with close proximity to the modiolus and thus to the spiral ganglion cells (Rebscher et al., 2008; Li et al., 2021).

### 2.7. ECAP thresholds

Intraoperatively, the Custom Sound EP software with its AutoNRT algorithm (Van Dijk et al., 2007) was used to measure T-ECAPs at all 22 electrode contacts. Stimulation and recording parameter settings were kept at default settings (stimulation rate, 250 Hz; stimulus and masker pulse width, 25 μs; stimulus-masker gap, 400 μs; sweeps, 35; gain, 50 dB). The starting level was set to 170 current units (CU). The algorithm uses the forward masking paradigm (Brown et al., 1990) in order to reduce the stimulus artifact. In general, AutoNRT increases the biphasic stimulation pulses in 6 CU steps until two reliable responses (N_1_ and P_1_) in a row were found; subsequent the stimulation levels decrease in 3 CU steps until a nonresponse is found. Then, the T-ECAP is estimated as the mean from the lowest recorded response and the highest recorded nonresponse (Van Dijk et al., 2007). The T-ECAP measurement was performed after impedance measurement and conditioning of all electrode contacts.

In postoperative T-ECAP measures, the conscious CI subject might show discomfort at certain stimulation levels and/or electrodes. If the “loudest acceptable presentation level” is reached before a reliable response was recorded, results will show an incomplete T-ECAP profile. By measuring T-ECAPs intraoperatively, we were able to collect data for all 22 intracochlear electrode contacts in all subjects.

### 2.8. Data analysis

Statistical analyses were performed using IBM SPSS Statistics for Windows, version 24 (IBM Corp., Armonk, N.Y., USA) and MATLAB™ software (The MathWorks, Inc., Natick, Massachusetts, USA).

Normal distribution (Shapiro–Wilk test) did not apply to all variables. Therefore, the nonparametric Friedman test and the Kruskal–Wallis test were used for comparing the equality of paired and unpaired samples, respectively. *Post hoc* analysis was performed and *p* values were adjusted by using the Bonferroni correction for multiple comparisons. Correlation analysis was performed by Spearman rank correlation. Statistical significance was defined as *p* < 0.05.

## 3. Results

### 3.1. PTA and speech perception

**Figure 3:**
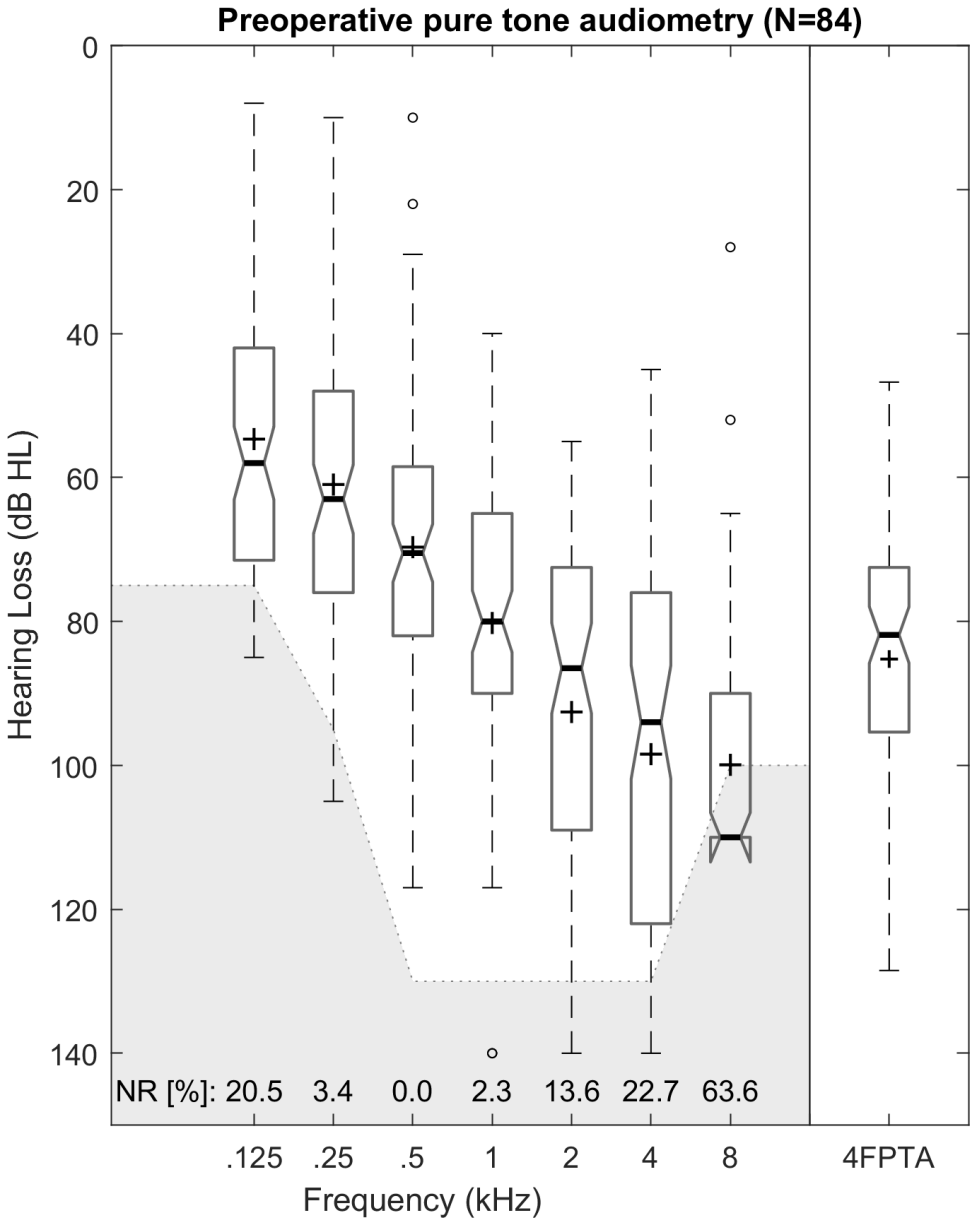
Box plot of unaided pure-tone audiometric threshold for octave frequencies from 0.125 to 8 kHz and pure-tone average of (4FPTA). Median values are indicated by thick lines, mean values by +, dashed lines illustrate the 25th and 75th percentiles, outliers are shown as circles. The greyed-out area marks the audiometers maximum output level. Number of “no response” (NR) is presented for each frequency in percent.

The mean preoperative hearing loss ranged from 55 dB HL (125 Hz) to 100 dB HL (8 kHz) and is illustrated in Figure 3 and Table 1. Differences among pure-tone frequencies were found to be significant [Friedman test: *χ*^2^(6) = 269.89, *p* < 0.001]. Median 4FPTA was 82 dB HL (SD 18 dB HL; range 47–129 dB HL).

Mean WRS_max_ was 35% (SD 26%; range 0–100%). WRS_max_ and 4FPTA were significantly correlated (*r* = −0.51, *p* < 0.001).

### 3.2. Insertion depth angle and electrode positioning

The cochlear parameters of the basal turn diameter A value (mean 9.0 mm; SD 0.5 mm) ranged from a minimum of 8 mm to a maximum of 10 mm. An average insertion depth angle θ_apical_ of 380° (SD 24.2°) was achieved, ranging from 315° to 425°. Mean insertion depth at the most basal electrode (θ_basal_) was 9.4° (SD 5.2°; range 1–25°). A negative correlation was found between the insertion depth angle θ_apical_ and the cochlear size with regard to diameter A (*r* = −0.33; *p* = 0.003).

The medial-lateral position MP ranged between 0.53 and 0.70 with a mean of 0.62 (SD 0.03). The most apical electrode E22 reached a mean SGC frequency of 749 Hz (SD 100 Hz) with a range between 615 and 1,162 Hz, while the most basal electrode E1 covered the SGC region around 16 kHz (SD 0.63 kHz; range 14.1–17.1 kHz).

### 3.3. T-ECAP

Intraoperative T-ECAP measurements were performed in all 84 subjects at all 22 electrode contacts. In 57 out of the 1,848 electrodes tested, no valid T-ECAP could be measured. The overall success rate was 96.9%. In 61 subjects (73%) all 22 T-ECAPs could be measured. In eleven and two subjects respectively, 21 and 20 T-ECAPs were still detectable. In four subjects each, 19 and 18 T-ECAPs were measured. There was one subject with only 16 and one with only 14 valid T-ECAPs.

**Figure 4:**
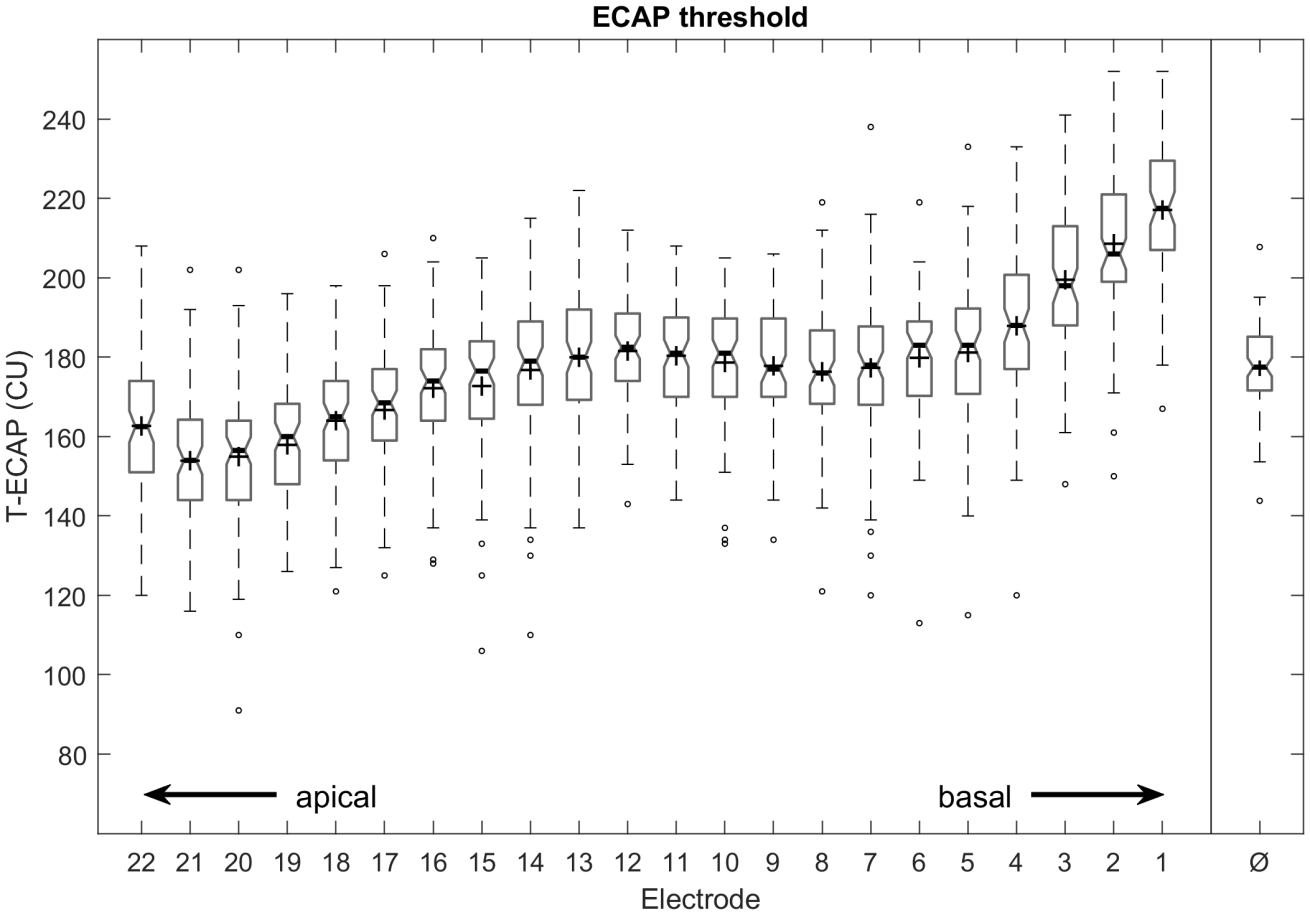
Intraoperative measured ECAP thresholds from most apical (E22) to most basal (E1) electrode contact number and averaged across all electrodes (Ø). Median values are indicated by thick lines, mean values by +, thin lines illustrate the 25th and 75th percentiles, outliers as circles.

The mean T-ECAP was 177 CU (SD 22 CU; range 91–252 CU). The mean T-ECAP profile shows a pattern of increasing T-ECAPs from apical to medial electrodes, reaching a plateau at the medial region before thresholds increased further toward the basal region of the electrode array (Figure 4). T-ECAPs differ significantly across the electrode array [Kruskal–Wallis test: *χ*^2^ (21) = 749.33, *p* < 0.001]. The pairwise comparison from *post hoc* analysis showed main differences among apical (e.g., EL20), medial (e.g., EL12) and basal (e.g., EL2) electrodes.

**Figure 5:**
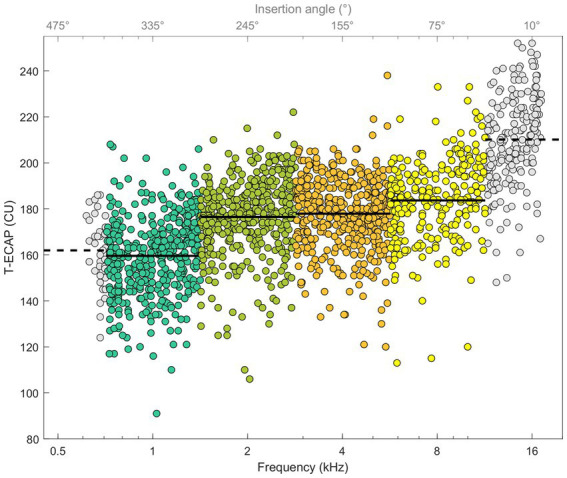
ECAP thresholds in relation to place of SG frequency (bottom x-axis) and insertion depth angle (top x-axis). Octave frequency bands 1, 2, 4, and 8 kHz with known hearing loss levels and T-ECAPs are colored. Solid line shows the average T-ECAP for each octave band. Frequency bands 0.5 and 16 kHz are shown with data points in grey and dashed line as T-ECAP average, but are not further evaluated.

Figure 5 illustrates ECAP thresholds in relation to their place of stimulation. The Kruskal–Wallis test showed differences between T-ECAP and place of stimulation [*χ*^2^(3) = 367.06, *p* < 0.001]. *Post hoc* comparison revealed significantly higher ECAP thresholds for electrodes within the SG octave frequency band 8 kHz compared with 1, 2 and 4 kHz. Conversely, T-ECAPs around 1 kHz were significantly lower compared with those at 2, 4 and 8 kHz.

### 3.4. ECAP and insertion depth

No correlation was found between the medial-lateral position MP and mean T-ECAP (*p* = 0.724). Also, the insertion depth angles θ_apical_ and θ_basal_ were not correlated with T-ECAPs at the corresponding EL22 (*p* = 0.539) and EL1 (*p* = 0.131).

There was no correlation found between cochlear diameter A and mean T-ECAP (*p* = 0.345).

### 3.5. Audiometric parameters and ECAP

**Figure 6:**
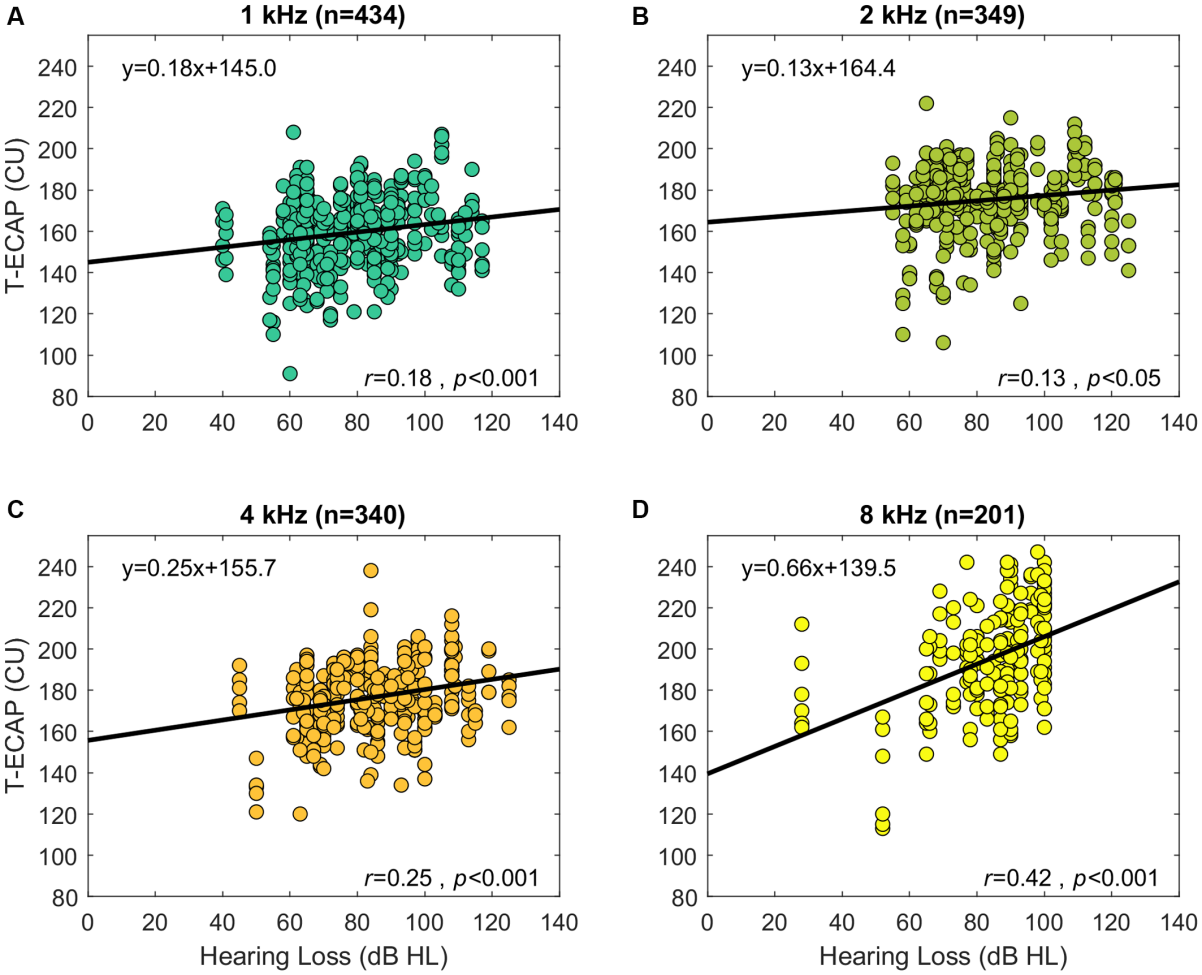
Preoperative audiometric thresholds relation to its corresponding ECAP threshold (abscissa). The panels show the hearing loss at SG-frequency 1 kHz **(A)**, 2 kHz **(B)**, 4 kHz **(C),** and 8 kHz **(D)** in relation to T-ECAPs. T-ECAPs and hearing loss were grouped according to the electrodes place of SG frequency and measurability of both T-ECAP and hearing loss. Sample size of each subgroup is given in brackets. The thick black line shows the result of linear regression analysis.

Hearing thresholds as represented by the 4FPTA and by T-ECAP were significantly correlated (*r* = 0.2, *p* < 0.05). Additionally, Figure 6 shows the significant correlation of hearing loss at single SG-frequency 1 kHz (*r* = 0.18, *p* < 0.001), 2 kHz (*r* = 0.13, *p* < 0.05), 4 kHz (*r* = 0.25, *p* < 0.001), and 8 kHz (*r* = 0.42, *p* < 0.001) with T-ECAPs at the corresponding placed electrodes. Linear regression analysis was performed for each subset.

No correlation was found between mean T-ECAP and WRS_max_ (*p* = 0.575). Also, there was no correlation found between cochlear diameter A and 4FPTA (*p* = 0.989).

## 4. Discussion

The main finding of this study was that ECAP values measured intracochlearly are quantitatively correlated with preoperative hearing. Similar results were reported for acoustically measured compound action potentials measured by electrocochleography (Walia et al., 2022). From the clinical perspective, ECAP thresholds may be used as a quality measure as they convey information about the nerve and the electrode–nerve interface immediately after implantation.

### 4.1. Preoperative audiometric data

Before cochlear implantation, audiometric measures are assessed in order to identify CI candidates. As Hoppe et al. (2019) pointed out, CI candidates with even substantial residual hearing on the side of CI implantation benefit from the CI. Since we conducted our investigation only in subjects with preoperatively measurable residual hearing, the mean 4FPTA of 82 dB HL in our cohort was lower than in earlier studies of subjects with Slim Modiolar electrodes (86–98 dB HL; Ramos-Macías et al., 2018; Hey et al., 2019; Liebscher et al., 2021). Hearing levels ranged in our study from 58 dB HL at the lower (125 Hz) to 110 dB HL at the higher (8 kHz) frequencies. This decline toward higher frequencies is typical for CI candidates with moderate-to-severe hearing loss (Hughes et al., 2014; Ramos-Macías et al., 2018; Hoppe et al., 2021). Sensorineural hearing loss is caused by changes in peripheral processing due to damage to the inner hair cells and/or the auditory nerve. This damage is generally more severe in the basal half of the cochlea than in the medial and more apical regions.

Pure-tone thresholds in CI candidates for frequencies above 6 kHz are usually beyond the technical limits of the audiometer (e.g., in this study 130 dB SPL at 6 kHz), indicating complete loss of function of the inner hair cells at and above that frequency those frequencies. Therefore, we could not measure and include frequencies beyond 8 kHz in our analysis; even though it can be expected that these basal regions are affected the most by auditory sensory loss and auditory deprivation.

### 4.2. Electrode insertion depth

We investigated only fully inserted electrodes with complete scala tympani insertion. The results of the average insertion depth angle θ_apical_ of 380 ± 24.2° is comparable to results of other studies using the Slim Modiolar electrode (Aschendorff et al., 2017) or comparable perimodiolar electrode arrays with similar length (Landsberger et al., 2015), in which θ_apical_ of 403° and 381° respectively were reported.

Aschendorff et al. (2017) additionally reported a slightly deeper insertion of the most basal electrode (θ_basal_) of 18° compared with the 9.4° found in this study. These variations can result from various factors, e.g., different surgical approaches (round window, extended round window or cochleostomy). The round window approach was more frequently used in this study (88% in our study vs. 44% in that of Aschendorff et al., 2017); an extension of the round window or cochleostomy can lead to an increased insertion depth angle (Briggs et al., 2006). Additionally, the cochlear anatomy is known to influence the achievable insertion depth angle, since a larger diameter A and cochlear duct length will reduce the overall possible insertion depth (Escudé et al., 2006; Franke-Trieger and Mürbe, 2015; Ketterer et al., 2018), even though the cochlear diameters A in both studies were in line (average of 8.9 vs. 9.0 mm). Lastly, Aschendorff et al. (2017) illustrated the influence of positioning of the “depth markers,” which provide assistance during surgery indicating the electrode array’s insertion depth in relation to the round window opening, to the actual achieved insertion depth and to the medial-lateral position. The best perimodiolar placement was achieved when the first marker was placed at the cochlea opening. The findings of Aschendorff et al. investigating the Slim Modiolar electrode were highly relevant for surgeons and their insertion approach with this electrode array later on.

The average medial-lateral position from this study of 0.62 matches the results of Aschendorff et al. (2017). The lower variation of 0.03 found in this study (compared with 0.05) and smaller maximum outliers of 0.7 (compared with 0.77) may be the result of the overall increase in experience with this electrode.

### 4.3. ECAP thresholds

Intraoperative T-ECAPs could be recorded reliably for all subjects. We had a high overall success rate in finding ECAP thresholds (96.9%). This success rate is even higher than the previously rate of 95.6% reported by Liebscher et al. (2021), who investigated different perimodiolar electrode arrays, and it is also higher than the 90% reported by Hey et al. (2019), who studied the Slim Modiolar electrode. We interpret our high success rate as resulting from the fact that we only investigated in correctly placed ST insertions and had no pathological cases. However, there are still some limitations due to the use of the AutoNRT algorithm in the intraoperative test setting. Intraoperatively, the main reasons for missing T-ECAPs are electrical compliance complications, in which no ECAP response are detected as the maximum current level could not be reached owing to increased impedances (Spivak et al., 2011; Hey et al., 2019). Additionally, even with pre-conditioning applied, recording artefacts can occur (Van Dijk et al., 2007) that lead to a response being missing. However, all electrodes and electrode contacts worked within the specifications; throughout the test there were no open- or short-circuits.

The average T-ECAP profile of perimodiolar electrodes has been demonstrated in numerous studies. The profile typically shows increasing ECAP thresholds from the apical to the medial electrodes, where a plateau or in some cases a maximum is reached, with a slight decrease before increasing further (Figure 4) toward the basal part of the electrode array (Spivak et al., 2011; Müller et al., 2015; Hey et al., 2019; Mewes et al., 2020; Liebscher et al., 2021). Interestingly, the Slim Modiolar electrode shows lower T-ECAPs at the apical electrodes than its “perimodiolar counterpart” the Contour Advance electrode (Liebscher et al., 2021). One reason for this is the much less frequent incidence of an electrode being translocated from ST into the SV (Aschendorff et al., 2017; Shaul et al., 2020; Liebscher et al., 2021), a displacement that is accompanied by significantly increased T-ECAPs (Mittmann et al., 2015; Venail et al., 2015; Liebscher et al., 2021).

Electrode impedance can also influence T-ECAPs. Venail et al. (2015) demonstrated that higher impedance leads to lower ECAP thresholds in postoperative measures, and they concluded that fibrotic tissue growth surrounding the electrode contacts changes the electrical current path and therefore the impedance value. However, since we only investigated intraoperative T-ECAPs measured right after the insertion, tissue growth should not be an issue. Nonetheless, if the contact surfaces of single electrodes are surrounded by different fluids or tissues, then the impedance values will differ. Both electrode types (Slim Modiolar and Contour Advance) show different impedances patterns (Mewes et al., 2020), since the Slim Modiolar electrode is significantly smaller in volume than the Contour Advance. Therefore, less space within the cochlea is used and the intracochlear electrodes’ positioning differs, which can lead to different current paths.

Regarding the electrodes’ intracochlear positioning, in our results the average medial-lateral position of 0.62 shows that there is an overall small distance between electrode contacts and the modiolus. Regardless of this, the physical electrode-to-modiolus distance is not correlated with ECAP thresholds, which confirms the findings of Venail et al. (2015).

Lastly, the Slim Modiolar electrode is designed to be more effective with regard to the preservation of residual hearing. Therefore, patient recruitment might have changed over time, since more subjects with substantial amount of residual hearing nowadays receive this perimodiolar electrode array instead a straight (e.g., CI622) or shorter (e.g., CI624) electrode array.

### 4.4. T-ECAP and preoperative hearing loss

The effects of reduced ECAP responses caused by a smaller amount of surviving SGCs have been shown in animal studies (Prado-Guitierrez et al., 2006; Ramekers et al., 2014). Ramekers et al. (2014) demonstrated in their histological and electrophysiological analysis that a reduced packing density of SGCs in the deafened group of guinea pigs led to a decreased ECAP amplitude growth function. The neural survival of the peripheral auditory system can be revealed in an objective manner by using ECAPs with CI Systems.

Our cohort of CI users showed a similar relationship. Subjects with better preoperative hearing also had lower ECAP thresholds, meaning that a smaller amount of electrical stimulation was needed to determine the objective auditory response. We therefore take this as further evidence that T-ECAPs can reflect intracochlear neuronal health status. This correlation is evident for frequencies 1, 2, 4 and 8 kHz (Figure 6). Since the hearing loss is generally less distinct in the apical than in the basal cochlear region, T-ECAPs are lower at the apical than at the basal electrodes. Our results support the findings of Nassiri et al. (2019), who also found lower T-ECAPs at apical electrodes in subjects with residual hearing. The relationship of decreasing ECAP thresholds between apical and basal regions can also be seen for the characteristic frequencies below 1 and beyond 8 kHz (Figure 5). However, since the average insertion depth (θ_apical_) of the most apical EL22 is 380°, which corresponds to 749 Hz (SD 100 Hz) SG frequency, there are only few subjects with the most apical electrode inserted within the octave band with the 500 Hz center frequency; therefore, this frequency group was not included in the final analysis. Additionally, sufficient hearing thresholds beyond 8 kHz were not encountered in any subject; hence, we could not include higher-frequency groups in our correlation analysis with ECAP measures.

Nonetheless, it is important to point out that around an SG frequency of 16 kHz the overall highest ECAP thresholds were found. With regard to our findings, we assume that in these basal regions the neural survival of the peripheral auditory system is particularly low. Studies that used histopathological analysis support this assumption. For example, Wu et al. (2021) recently examined the survival rate of inner and outer hair cells, and of peripheral axons of the auditory nerve fibers, from temporal bones in normal-hearing and noise-exposed human subjects. They found an age-related loss in hair-cell and peripheral-axon counts in both groups in the apical and basal regions. However, the noise-exposed group showed smaller counts for high frequencies and lower hearing thresholds. Additionally, studies investigating the primary neuronal loss by counting SGC in temporal bones of hearing-impaired human subjects show a negative correlation with word-recognition scores and audiometric thresholds (Otte et al., 1978; Sagers et al., 2017). Makary et al. (2011) also displayed the age-related decline in SGC counts in healthy subjects. Their findings showed also a trend of increased degeneration in the basal turn compared to the upper turns.

Overall, histopathological studies show that auditory neuronal loss occurs at various stages (hair cells, SGCs) and is driven by age, etiology and severity of the hearing impairment.

Lastly, we found no correlation between intraoperative mean T-ECAP and preoperative WRS_max_. The arithmetic T-ECAP mean of all electrodes might be too vague, since speech recognition requires central auditory processing and T-ECAPs do not provide information about temporal processing. Other study groups who investigated in the relationship between ECAP measures and postoperative speech perception outcome with CI showed a more complex picture. Instead of just using T-ECAPs, Skidmore et al. (2021) generated an ECAP index value that consists of various different ECAP measures, including T-ECAP, slope of the amplitude growth function, N_1_-latency and refractory recovery function. In adults, they showed a significant correlation of Consonant-Nucleus-Consonant-word (CNC) lists and AzBio sentence measures in quiet with the ECAP index. He et al. (2022) showed that subjects with a prolonged speed of recovery from neural adaptation tend to have poorer speech perception outcome. Similar findings were shown by another publication of Skidmore et al. (2023), who additionally found significant correlation of the ECAP index with CNC words and AzBio sentences in noise. These studies show, that in order to find a correlation of auditory performance with ECAP measures, a more extensive analyses from the characteristics of neural response is needed.

### 4.5. Diagnostic value and limitations

In CI candidates with preoperative speech perception, WRS_max_ can be a predictor of the minimum speech perception obtained with CI later on (Hoppe et al., 2019). In subjects without speech perception, pure-tone thresholds are still useful in order to estimate neuronal health status.

It was shown recently (Fontenot et al., 2019; Kim, 2020), that electrocochleography correlates with auditory perception. When using acoustic stimulation ECoG measurements are influenced by both the hair cell functioning and the neural processing. For electrically evoked responses as used in our study no acoustic-electric conversion is necessary. Hence, ECAP deliver information on the neural components without being influenced by hair cell functioning.

There are still factors which are not recorded but might affect the correlation in our findings. E.g., in cases of retrocochlear hearing loss at certain frequencies; there are no valid hearing levels, but one might find peripheral responses (T-ECAPs). Additionally, we have no knowledge of the individual existence of potential cochlear dead regions (Moore and Malicka, 2013). If there are non-functioning inner hair cells at a certain region for a specific frequency, adjacent hair cells may evoke neural excitation. Due to this “off-place listening” the measured pure-tone audiometric thresholds might not reflect the actual hearing loss properly.

A similar effect might occur at the electrophysiological level in ECAP measures. Due to the monopolar stimulation mode, the spatial spread generated by the electrical stimulus of one single electrode can cause excitation of neighboring SGNs (Cohen et al., 2003). It has been shown, that current focused stimulation modes can provide less current spread (Bonham and Litvak, 2008; Padilla and Landsberger, 2016), providing a more place-specific electrical stimulation. However, this technique is not yet available in standard (T-)ECAP measures.

## 5. Conclusion

This study in cochlear implant (CI) users with preoperative residual hearing analyzed the relationship of peripheral health status on electrically evoked action potentials with CI. In our study cohort, a significant link was found between preoperative pure-tone hearing levels and objective hearing-nerve responses at the corresponding stimulation site.

This new insight adds another element in the complex relationship of the various factors that influence the subsequent outcome with a CI.

## Data availability statement

The raw data supporting the conclusions of this article will be made available by the authors, without undue reservation.

## Ethics statement

The studies involving humans were approved by Ethikkommission der Friedrich-Alexander-Universität Erlangen-Nürnberg. The studies were conducted in accordance with the local legislation and institutional requirements. Written informed consent for participation was not required from the participants or the participants’ legal guardians/next of kin in accordance with the national legislation and institutional requirements.

## Author contributions

The study was designed by TL and UH. TL, UH, and JH collected the data. TL analyzed and interpreted the data. The manuscript was written by TL. All authors read and approved the final manuscript.
